# Integral Design Methodology of Photocatalytic Reactors for Air Pollution Remediation

**DOI:** 10.3390/molecules22060945

**Published:** 2017-06-07

**Authors:** Claudio Passalía, Orlando M. Alfano, Rodolfo J. Brandi

**Affiliations:** 1 Facultad de Ingeniería y Ciencias Hídricas, Universidad Nacional del Litoral and CONICET, Santa Fe 3000, Argentina; cpassalia@unl.edu.ar; 2 Instituto de Desarrollo Tecnológico para la Industria Química (CONICET-UNL), Santa Fe 3000, Argentina; rbrandi@santafe-conicet.gov.ar

**Keywords:** air pollution, photocatalytic reactors, radiation modeling, reactor optimization

## Abstract

An integral reactor design methodology was developed to address the optimal design of photocatalytic wall reactors to be used in air pollution control. For a target pollutant to be eliminated from an air stream, the proposed methodology is initiated with a mechanistic derived reaction rate. The determination of intrinsic kinetic parameters is associated with the use of a simple geometry laboratory scale reactor, operation under kinetic control and a uniform incident radiation flux, which allows computing the local superficial rate of photon absorption. Thus, a simple model can describe the mass balance and a solution may be obtained. The kinetic parameters may be estimated by the combination of the mathematical model and the experimental results. The validated intrinsic kinetics obtained may be directly used in the scaling-up of any reactor configuration and size. The bench scale reactor may require the use of complex computational software to obtain the fields of velocity, radiation absorption and species concentration. The complete methodology was successfully applied to the elimination of airborne formaldehyde. The kinetic parameters were determined in a flat plate reactor, whilst a bench scale corrugated wall reactor was used to illustrate the scaling-up methodology. In addition, an optimal folding angle of the corrugated reactor was found using computational fluid dynamics tools.

## 1. Introduction and Scope

Indoor air quality became a generalized concern in the last few decades. People spend most of their time in confined environments and may be exposed to a poor air quality for extended periods of time. Such a situation is regarded as a human health concern [1,2,3]. The effects in human health of a poor air quality in a room can range from headaches to nausea, dizziness, eye and nose irritations, dry cough and tiredness [4], a situation known as sick building syndrome (SBS). This syndrome is usually associated with the presence of volatile organic compounds (VOCs) in very low concentrations [1,5,6].

A poor indoor air pollution quality may result from in situ generation of compounds or from an exchange with the outside. For many pollutants, it is usual to find indoor concentrations larger than outdoors, particularly VOCs, which are emitted from building materials, furniture and equipment [4]. Among indoor VOCs, simple aldehydes such as formaldehyde (HCHO) are typically found in polluted places [7].

The recognition of the health effects of indoor VOCs implies the need to attain their depletion. The reduction of pollutant concentrations in air has been traditionally centered in the source control, the increasing of air renewal rates and the application of air cleaning devices. Conventional control processes usually employed are filtration and adsorption; these technologies present certain drawbacks, but above all they require final disposal because there is a transfer of the pollutant from the gas phase to a solid one. In this context, indoor air quality may be controlled by heterogeneous photocatalysis, an effective alternative to conventional technologies that has been tested to chemically destroy a large variety of airborne pollutants [8,9,10].

In the photocatalytic process, the compounds present in the air stream may be adsorbed onto the surface of a catalyst, upon which the irradiation starts a series of superficial reactions that can lead to the chemical degradation of pollutants. Although the final products of organic pollutants containing no hetero-atoms are water and carbon dioxide, numerous intermediate products may appear in the reaction steps and their elimination must also be taken into account [11].

Photocatalytic reactors must gather the molecules of the pollutant, the surface of the photocatalyst particles and the radiation energy in the proper wavelength at once. Thus, the design and modeling of such reactors presents the need for solving the radiation field in addition to the classical momentum, energy and species mass balances.

The aim of this work is to present a methodology for an integral design of photocatalytic reactors for the control of air pollution as a synthesis and review, including the development of intrinsic kinetic models [12], reactor scaling-up [13] and optimization [14]. The work is organized in two main sections, being the first a detailed description of the proposed methodology and the second a concrete and successful application.

## 2. Integral Design Methodology

**Figure 1:**
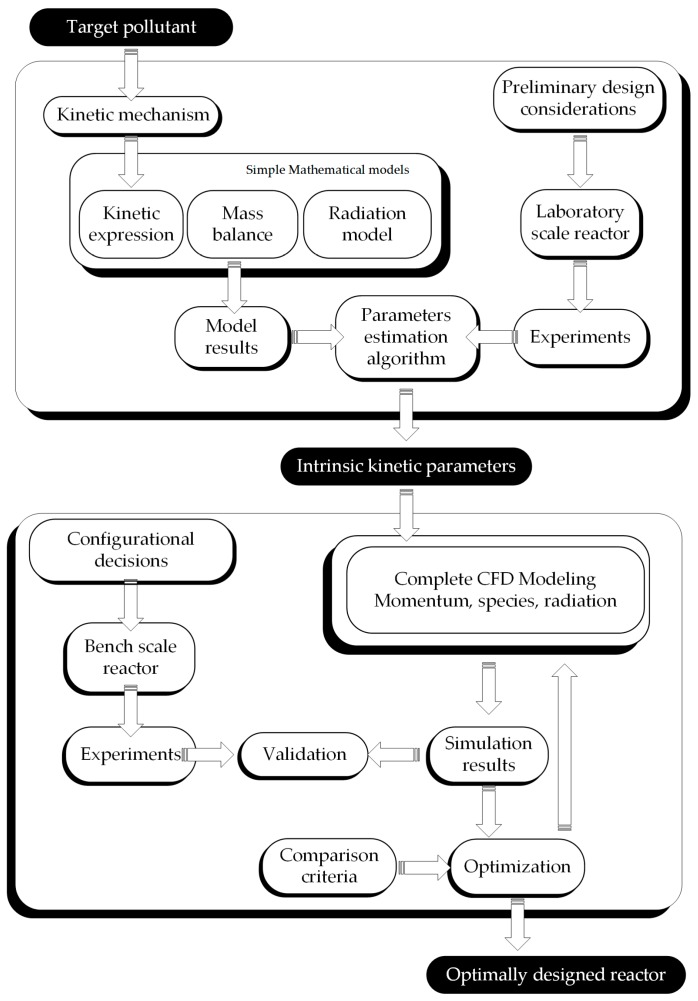
Conceptual framework of the integral design methodology.

The proposed methodology for the complete procedure of integral reactor design is schematically represented in Figure 1, and may be summarized in the following steps.

### 2.1. Kinetic Study

There are many chemical compounds usually found in polluted indoor air, among which the family of VOCs can be found. The selection of one or more pollutants to be eliminated is generally the first step in a photocatalytic study. Every compound, with its own physicochemical properties, has its implications regarding the experimental issues, such as generation and analytical determination. Then, a kinetic mechanism for the photocatalytic degradation of the selected target compound can lead to obtaining an analytical reaction rate expression. This rate expression must include the dependence on the pollutant and stable intermediates concentrations and the local superficial rate of photon absorption (LSRPA or $e^{a,s}$) [15].

To carry out experimental tests in photocatalysis, it is important to consider, previously to the reactor design, aspects such as the photocatalyst’s characteristics (its optical properties, its chemical stability or its deactivation), the support material in which the catalyst is to be fixed, the radiation source (emission spectrum and power) and the interactive radiation in the interior of the reactor.

The selection of the catalyst is one of the essential steps to be considered in the design and application of a photocatalytic reactor [16]. In general, photocatalysts are metal oxides that behave as semiconductors which absorb radiation in a certain wavelength range. Titanium dioxide (TiO_2_) is the most widely used photocatalyst because of its chemical stability, low toxicity and relatively low cost. TiO_2_ absorbs radiation in the ultraviolet (UV) range of the spectrum to promote electrons across the energy gap into the conduction band. The vacancies or holes left by those electrons may then form hydroxyl radicals capable of attacking adsorbed organic compounds onto the catalyst’s surface.

The depuration of polluted air requires the fixation of TiO_2_ over some material acting as a support, given that the catalyst should not be dragged by the gas stream. Among the possibilities of immobilization on the support material one can find photocatalyst coatings, layers and films (but they can also be dispersed in a matrix to build monoliths). The techniques to affix the catalyst and support mainly include variants of dip-coating and sol-gel methods. The purpose is to obtain the largest possible surface area to volume ratio, a large area exposed to radiation and good adherence to an inert substrate. Materials tested to act as inert supports are diverse: glass, metals, fibers, plastics, etc. [17].

The radiation that initiates the superficial phenomena leading to the pollutant elimination may be artificial or natural, i.e., coming from a lamp or from the Sun. According to which source of radiation will be used, the geometry of the reactor may differ greatly. Because of the TiO_2_ bandgap, it cannot profit from a large portion of the solar spectrum [18]; thus, efforts are constantly directed towards the doping of TiO_2_ for extending the absorption at larger wavelengths (>390 nm) towards the visible range. Doping with C, N, Ce, etc. has been studied with certain success [19,20].

Regarding the size and configuration of the laboratory scale reactor, simplicity is desirable. A simple geometry reactor ensures the application of a simple mathematical model and, under selected operating conditions, the absence of mass transfer limitations. In addition, a uniform radiation flux allows the incorporation of the LSRPA as a constant in the kinetic model. Typically, the radiation model is based on an emission model for the lamps [15] and the determination of spectral optical properties of the catalyst layer and support. In accordance to what has been said above, it is clear why the flat plate reactor may be thought of as a standard for kinetic studies: under the imposed size, geometry and operation, it can be accurately modeled as a plug flow reactor with a straightforward solution.

Thus far, we have a simple reactor model, coupled with the kinetic expressions and mathematically solved. The experimental data from the laboratory reactor together with the mathematical model allow the determination of the unknown kinetic parameters. The procedure for estimation of the kinetic parameters is typically achieved by running a numeric nonlinear algorithm to fit the model predictions to the experimental data. An intrinsic kinetics implies the independence of reactor design variables, including the radiation source and operating variables. In this sense, two important considerations need to be satisfied for the application of the kinetic parameters obtained in the kinetic reactor to the scaling up: (i) to employ the same catalyst and immobilization technique in the larger scale reactor; and (ii) to ensure that the experimental data in the laboratory reactor are obtained under kinetic control regime to eliminate the effect of mass transfer limitations.

### 2.2. Scaling and Optimization Methodology

As previously stated, the present work addresses the implementation of a methodology for the scaling-up and optimization of a photocatalytic reactor using experimental data obtained in a simple geometry laboratory scale reactor. As depicted in Figure 1, for scaling-up purposes (changing the reactor shape, size, lamps, and configuration), the kinetic model developed in a previous step is directly applied to the new reactor. The solution of the mass balance and the radiation model allows predicting the performance of the bench scale reactor.

Once the explicit reaction rate and the kinetic parameters are known, their application to any reactor size or configuration is direct, provided that: the mass balances in the reactor can be solved and the radiation field can also be evaluated properly. Thus, the LSRPA must be known in the new reactor which may differ from the laboratory one not only in size or shape but also in its radiative behavior. The obtained field of LSRPA is introduced in the mass balance to simulate the reactor performance and predict its conversion.

When it comes to bench scale reactors, the biggest modifications are related to the inner and outer configuration and size, i.e., the geometrical arrangement of the radiation source with respect to the reaction space; the operation regime (continuous flow, batch, recycle, etc.); and the inner reactor geometry (shape and dimensions).

Photoreactor configuration and geometry are essential because, in addition to the velocity and concentrations profiles, radiation field must be known for a rigorous modeling. As has been previously stated, for the treatment of polluted air streams, the catalyst is immobilized on the support, regardless of the reactor type. In this respect, the best available option is the family of photocatalytic wall reactors. Among the geometries or configurations of photocatalytic wall reactors one can find: the flat plate reactor [12,21], the multi-plate reactor [22], monolith and honeycomb reactors [23,24], mesh reactors [25,26], annular or multi-annular reactor [27,28], optical fiber [29] and corrugated [30,31] reactors, all of which were applied with acceptable to good efficiency in the abatement of innumerable compounds.

The following step is validation, where the data obtained from experimental runs in the bench scale reactor are contrasted against simulation results. Photocatalytic reactors entail simultaneously momentum, mass and radiation transfer. Their design usually needs an optimization step in order to obtain the best global performance possible. The comprehensive mathematical simulation of photochemical reactors is a very helpful and affordable tool nowadays with computational capabilities sufficiently large to provide a detailed approach to all phenomena involved. In particular, computational fluid dynamics (CFD) tools have been increasingly used to model very different kinds of processes, including the modeling of gas phase photocatalytic reactors [31,32,33].

## 3. Application: Step-by-Step Design and Optimization of a Photocatalytic Reactor

### 3.1. Laboratory Scale Reactor

Recalling Figure 1, the determination of kinetic parameters is based on experimental data, in particular, the assays performed in the laboratory scale reactor.

#### 3.1.1. Experimental

**Figure 2:**
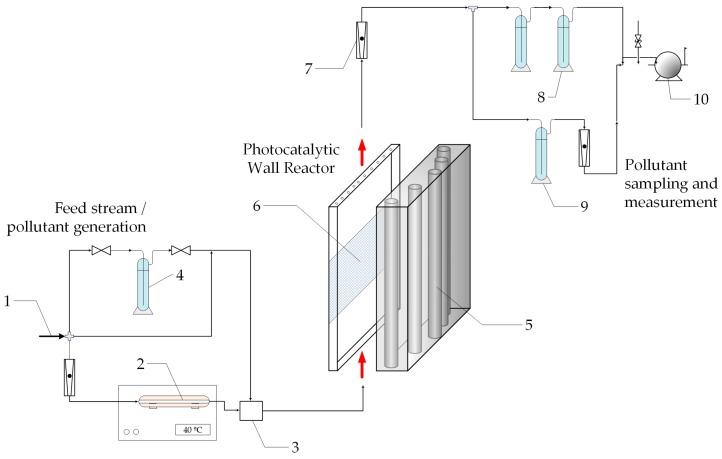
Experimental set-up: (1) Clean dry air; (2) thermostated formaldehyde (HCHO) generator; (3) mixer; (4) water vapor saturator; (5) ultraviolet (UV) lamps; (6) TiO_2_ coated stainless steel plate; (7) variable area flowmeter; (8) scrubbers; (9) HCHO collection; and (10) pump.

A complete experimental set-up was designed and constructed; a scheme is presented in Figure 2. The reaction device itself is a continuous, single-pass flat plate reactor with a photocatalytic wall [12].

The reactor consists of an acrylic parallelepiped; in its interior, the photocatalytic wall is a flat stainless steel plate coated with TiO_2_. The reactor window is transparent to radiation of wavelengths in the near UV range (300–400 nm). The radiation source is a group of five black light fluorescent lamps (Sylvania F15W T12) that provide a uniform radiation flux over a wide area (Table 1). This is achieved by a convenient arrangement of the lamp positions in relation with the reactor window, as seen in Figure 2.

The fluid feed to the reactor has a known pollutant concentration (formaldehyde) and relative humidity. The pneumatic circulation within the device is provided by a vacuum pump from its output; the volumetric flow rate is measured using variable area flowmeters. At the reactor outlet, samples are taken to determine the overall pollutant conversion.

#### 3.1.2. Procedures

*Catalyst preparation*: A flat plate of AISI 316 stainless steel (Acerind S.C., Santa Fe, Argentina) is the support material. TiO_2_ was fixed by cycles of impregnation with an aqueous suspension. A solution of methanol in water 25% (*v*/*v*) is prepared and pure TiO_2_ powder (Aeroxide P25 (Evonik Industries AG, Essen, Germany)) is then added to reach a 45 g/L concentration. Dip coating is performed in a vessel; after extraction, the piece is dried in stove and the cycle is repeated four times. The obtained layers of catalyst presented good uniformity and adherence to the metal support.

*Analytical techniques*: HCHO was generated online by heating solid paraformaldehyde. For the analytical determination of HCHO concentration in air, the experimental device was adapted to apply a colorimetric method.

*Experimental runs*: The experimental set-up was designed to collect samples only at the exit of the reactor; thus, the inlet HCHO concentration is established by taking samples over time with the UV lamps occluded. When a steady state inlet concentration is achieved, the lamps are uncovered and samples are taken in this irradiated period until the HCHO concentration reaches a new steady state, the outlet concentration. Different radiation levels were obtained using grey filters, which provided attenuations to give 16%, 26% and 60% of the maximum radiation flux.

#### 3.1.3. Kinetics

The starting point for this study is a simplified kinetic scheme based on a published reaction pathway [34]. After some algebraic work and the application of the micro steady-state approximation (MSSA), an analytical expression for the reaction rate was developed [12]. Table 2 presents the reaction scheme with all the involved steps:

Ending reaction 8 in Table 2 is a generic termination step for the active oxidizing species, where M is an inert species or body that can consume hydroxyl radicals. Formic acid (HCOOH) is an intermediate product that has a fast rate of disappearance compared to its formation rate and presents concentrations three orders of magnitude lower than those of HCHO [6]. In addition, a balance for adsorption sites on the catalyst surface for HCHO and water makes it possible to relate surface to bulk concentrations. The final reaction rate expression is:

(1) $$r_{F}=r_{5}=-\alpha_{1}\frac{\left(-1+\sqrt{1+\alpha_{2}r_{g}}\right)C_{F}}{1+\kappa_{W}C_{W}+\kappa_{F}C_{F}}$$

This four-parameter expression is a function of HCHO concentration ($C_{F}$), the water vapor concentration ($C_{W}$) and the superficial rate of electron–hole pair generation ($r_{g}$). The kinetic parameters $\alpha_{1}$ and $\alpha_{2}$ are combinations of kinetic constants, concentrations of species that remain inalterable or considered in excess, while $\kappa_{F}$ and $\kappa_{W}$ are adsorption equilibrium constants that relate bulk to surface concentrations. The local superficial rate of electron–hole pair generation can be defined as follows [15]:

(2) $$r_{g}(\underline{x})\;=\;\int_{\lambda }\Phi_{\lambda }e_{\lambda }^{a,s}(\underline{x})d\lambda \;=\;\bar{\Phi }\sum_{\lambda }e_{\lambda }^{a,s}(\underline{x})\;=\;\bar{\Phi }\cdot e^{a,s}(\underline{x})$$

where $e_{\lambda }^{a,s}$ is the LSRPA, a function of position ($\underline{x}$) and wavelength (λ), and $\bar{\Phi }$ is a wavelength averaged primary quantum yield. Experimental evidence of the linear dependence of the reaction rate with the radiation level was also found. Thus, the rate expression was simplified to give:

(3) $$r_{F}\;=\;\frac{-\alpha e^{a,s}C_{F}}{1\;+\;\kappa_{w}C_{w}\;+\;\kappa_{F}C_{F}}$$

where α is the main kinetic parameter.

#### 3.1.4. Modeling

As stated in Figure 1, the laboratory scale reactor must ensure the simplicity in the mathematical modeling. Considering the advantages of the simple geometry, the mass balance for formaldehyde in the reactor becomes an ordinary differential equation:

(4) $${\langle v_{z}\rangle }_{A_{c}}\frac{d{\langle C_{F}\rangle }_{A_{c}}}{dz}\;=\;a_{v}\;r_{F}$$

(5) $$z=0\;{\langle C_{F}\rangle }_{A_{c}}=C_{F}^{in}$$

where $a_{v}$ is the external catalytic surface area per unit volume and $r_{F}$ is the heterogeneous rate of disappearance of HCHO. Equation (4) represents the variation of the pollutant concentration along the reactor length due to the photocatalytic reaction. After inserting Equation (3) into Equation (4) and solving together with its boundary condition (Equation (5)) we have:

(6) $$\kappa_{F}\left(C_{F}^{out}-C_{F}^{in}\right)\;+\;\left(1+\kappa_{w}C_{w}\right)\ln\left(\frac{C_{F}^{out}}{C_{F}^{in}}\right)\;=\;-\alpha \frac{A_{cat}}{Q}e^{a,s}$$

This expression is implicit in terms of the cross-section averaged outlet formaldehyde concentration: $C_{F}^{out}={\langle C_{F}\rangle }_{A_{c}}(z=L)$. It was solved by means of a non-linear optimization procedure based on the Levenberg–Marquardt algorithm coupled with the set of experimental data to obtain the values of the three kinetic parameters.

*Radiation field model.* The evaluation of the LSRPA inside the reactor is based on the extense source with superficial emission (ESSE) model for the UV lamps [15] and the ray tracing method. This allows the integration of radiation contributions coming from any point at the lamps surfaces that are visible from the point of incidence considered on the catalytic film. The ESSE model is:

(7) $$I_{\lambda ,L_{i}}(y,z,\phi ,\theta )=T_{\lambda ,Wi}\;T_{\lambda ,Fi}\;I_{\lambda ,L_{i}}^{0}=T_{\lambda ,Wi}\;T_{\lambda ,Fi}\frac{P_{\lambda ,L_{i}}}{2\pi^{2}R_{L_{i}}L_{L_{i}}}$$

where $I_{\lambda ,L_{i}}^{0}$ is the radiation intensity leaving the source and computed using the superficial lamp emission model of the cylindrical actinic lamp. Here, $P_{\lambda ,L_{i}}$ is the emission power output of the lamp for a given wavelength provided by the manufacturer, and $R_{L_{i}}$ and $L_{L_{i}}$ are the lamp radius and length, respectively. The spectral relative emission power of the lamp and the acrylic window transmittance need to be measured.

Apart from that, for this multilamp system, the additive effect of each lamp “*i*” on the LSRPA must be taken into account. The expression of the LSRPA ($e^{a,s}$) at each point on the catalytic plate is:

(8) $$e^{a,s}(y,z)=\sum_{\lambda }\eta_{\lambda ,abs}T_{\lambda ,Wi}\;T_{\lambda ,Fi}\sum_{i}\;\int_{\phi_{1,L_{i}}}^{\phi_{2,L_{i}}}\int_{\theta_{1,L_{i}}}^{\theta_{2,L_{i}}}\frac{P_{\lambda ,L_{i}}}{2\pi^{2}R_{L_{i}}L_{L_{i}}}\sin^{2}\theta \;\sin\phi \;d\theta \;d\phi$$

where $\eta_{\lambda ,abs}$ is a radiation absorption fraction that can be defined as the ratio of absorbed to incident radiation; and $\phi_{1,L_{i}},\;\phi_{2,L_{i}},\;\theta_{1,L_{i}},\;\theta_{2,L_{i}}$ are the limiting angles from which radiation can reach the incidence point for the lamp i. The absorption fraction can be determined from spectral total reflectance measurements. Equation (8) represents the contribution of every lamp i to the superficial radiation absorption over the useful wavelength range of the lamp emission.

#### 3.1.5. Kinetic Parameters Estimation

In order to obtain intrinsic parameters for the degradation kinetics, the experimental data need to be obtained under kinetic control regime, i.e., in the absence of external mass transfer limitations. This was tested for our runs by the application of the following criterion that relates the observed reaction rate $r_{obs}$ and the mass transfer of the pollutant from the bulk to the catalyst surface [35]: $r_{obs}/k_{m}C_{F}^{in}<0.1$; in this expression, $k_{m}$ is a mass transfer parameter estimated through empirical correlations and $C_{F}^{in}$ is the inlet HCHO concentration.

**Figure 3:**
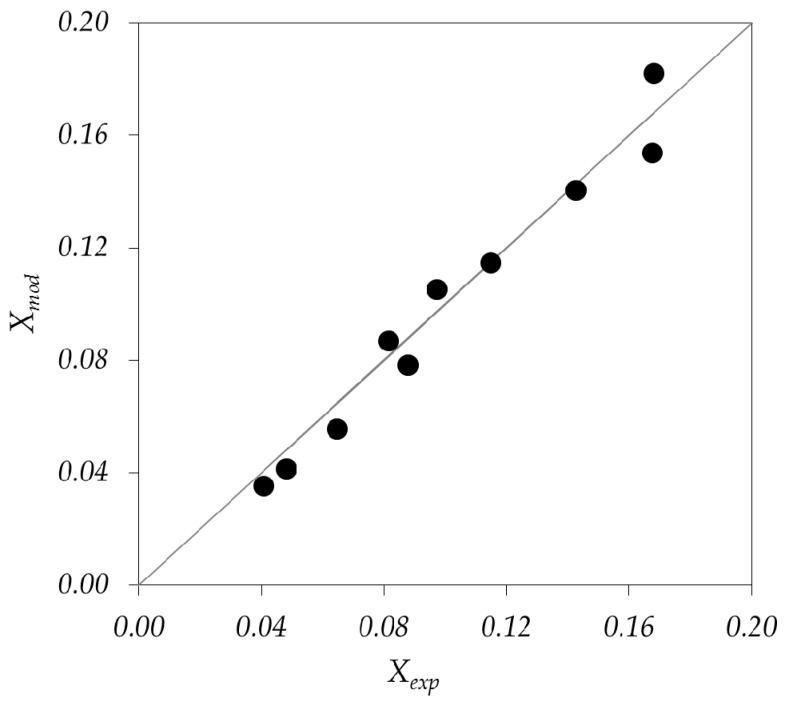
Results of the kinetic parameters estimation. Predicted pollutant conversion vs. experimental conversion (dots). The straight line represents a perfect fit. *X_mod_*: Pollutant conversion predicted by the model; *X_exp_*: experimental pollutant conversion.

The optimization procedure to obtain the values of the kinetic parameters in Equation (6) was a nonlinear algorithm that used the matrix of experimental data containing: (i) inlet HCHO concentration; (ii) relative humidity; (iii) radiation level; and (iv) outlet HCHO concentration. The values and units of the estimated parameters are: α = 1.34 × 10^8^ cm^3^ einstein^−1^; $\kappa_{F}$ = 7.17 × 10^9^ cm^3^ mol^−1^ and $\kappa_{W}$ = 2.97 × 10^6^ cm^3^ mol^−1^. The comparison between predicted and experimental HCHO conversions proved to be satisfactory as it can be seen in Figure 3.

### 3.2. Bench Scale Reactor: Corrugated Plate Reactor

Following with the procedure schematically shown in Figure 1, at this point we have a validated kinetics for the elimination of HCHO. The kinetic parameters are independent of radiation flux, so they can be applied directly to any reactor size and shape.

#### 3.2.1. Experimental Set-Up and Procedures

**Figure 4:**
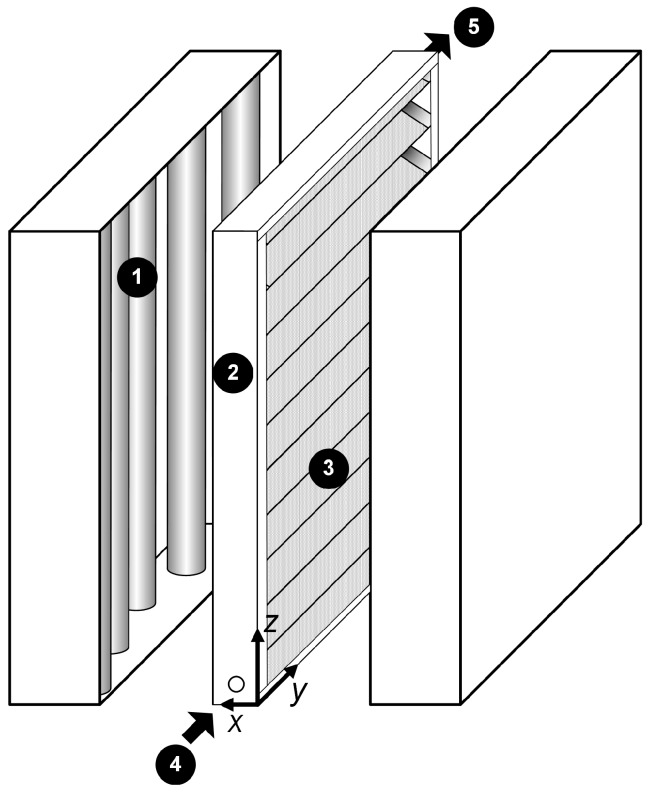
Corrugated wall reactor: (1) UV lamps; (2) acrylic frame; (3) stainless steel corrugated plate coated with TiO_2_; (4) reactor inlet; and (5) reactor outlet. Adapted with permission from Passalía, C.; Alfano, O.M.; Brandi, R.J. A methodology for modeling photocatalytic reactors for indoor pollution control using previously estimated kinetic parameters. *J. Hazard. Mater.* **2012**, *211–212*, 357–365. Copyright 2012 Elsevier [13].

The complete experimental set-up and procedures, except for the photoreactor itself, were the same as described in Section 3.1. The support of the bench scale photocatalytic reactor was also made of stainless steel; the stainless steel plate was successively folded into a one radian angle giving a total of 17 complete triangular section channels with their walls coated with TiO_2_ and placed inside an acrylic frame [31]. The reactor is irradiated by the same arrangement of black light lamps, but in this case from both sides of the reactor (Figure 4).

The corrugated reactor operates in continuous mode; air flows in a zigzag pattern from one triangular channel to another. Inlet pollutant concentration, water vapor concentration, radiation flux and total flow rate are fixed for each run.

By folding the metal plate, it is possible to obtain a more efficient use of available space and radiation, increasing the ratio between catalytic area per unit volume of reactor compared to a flat plate reactor under the same operating conditions. In addition, due to the possibility of interaction between catalytic walls by radiation reflection, one may expect the existence of an optimum angle: at more closed angles, the greater the reflective interaction between walls, the smaller the incoming radiation from the window.

#### 3.2.2. CFD Modeling

CFD packages allow the numerical, iterative and simultaneous solution of the governing equations of motion and reaction in a certain domain by volume discretization. The fundamentals of modeling the present system are: a reactive isothermal system with heterogeneous reaction; air is considered a Newtonian incompressible fluid with constant physical properties; and the process is under steady state and the flow regime is laminar. According to these conditions, the CFD model implies the resolution of the following set of differential equations:

Continuity equation:

(9) $$\underline{\nabla }\cdot \left(\rho \underline{v}\right)=0$$

Momentum equations:

(10) $$\underline{\nabla }\cdot \left(\rho \underline{v}\underline{v}\right)=-\underline{\nabla }P+\underline{\nabla }\cdot \left(\underline{\underline{\tau }}\right)+\rho \underline{g}$$

Conservation equations for species:

(11) $$\underline{\nabla }\cdot \left(\rho \underline{v}C_{i}\right)=-\underline{\nabla }\cdot {\underline{J}}_{j}+R_{i}$$

In Equations (9)–(11), ρ is the density, $\underline{v}$ the velocity, P the pressure, $\underline{\underline{\tau }}$ the viscous stress tensor, g the gravitational acceleration, $C_{i}$ the molar concentration of species i, ${\underline{J}}_{j}$ the diffusion flux vector and $R_{i}$ is the rate of production or depletion of species i by the homogeneous chemical reaction. The energy balance equation is not included provided that isothermal operation is assumed, with a fixed temperature of 298 K. In the system under study, there are no homogeneous reactions. Consequently, the term $R_{i}$ is zero for every species *i*. The calculated Reynolds number within the reactor was 140, so the laminar model was employed. The Newton’s law of viscosity is combined with Equation (10) as a constitutive equation for the definition of the stress tensor.

The approach for modeling the radiative interchange between windows and catalytic walls is by the introduction of user defined functions (UDFs). The incident radiation on the reactor windows was evaluated with the same approach to that of the flat plate reactor, i.e., based on the ESSE model for the lamps and the ray tracing method. The radiative interchange between windows and catalytic walls was also modeled externally and introduced as UDFs.

The radiation interchange can be modeled as follows: the reactor window is considered an emitting surface, while the catalytic walls are opaque and therefore can only reflect and absorb radiation. The radiation reflection on the catalyst surface is considered diffuse. At any position over the catalytic surface, radiation may come directly from the window or indirectly by reflection on the opposite catalytic surface.

The approach for modeling this radiative interaction is the surface to surface model by means of view factors [31,36]. Each surface is divided into small rectangular flat elements to compute de LSRPA. Elements in the same plane do not interact; in addition, the absorbed and reflected fractions do not depend on the position. The matrix of interaction between differential surface elements is solved numerically and the complete field of LSRPA over the corrugated plate is obtained.

#### 3.2.3. Simulation Results and Validation

The CFD resolution was performed with Fluent 12.1 (ANSYS Inc., Canonsburg, PA, USA). The pressure based segregated solver was used in a 3D model, under laminar flow regime and time-independent mode. The LSRPA was introduced in the suite as a custom function as well as the heterogeneous reaction rate expression (Equation (3)). In this way, the software solves the species balances subject to the imposed boundary conditions over the folded plate.

The 3D mesh was built with tetrahedrons and wedges and tested for grid independence. After a converged solution was obtained, the complete velocity, radiation and pollutant concentration fields inside the reactor were analyzed. The criteria of convergence were: scaled residuals decreased five orders, stabilized macroscopic monitors and overall mass balance conservation.

**Figure 5:**
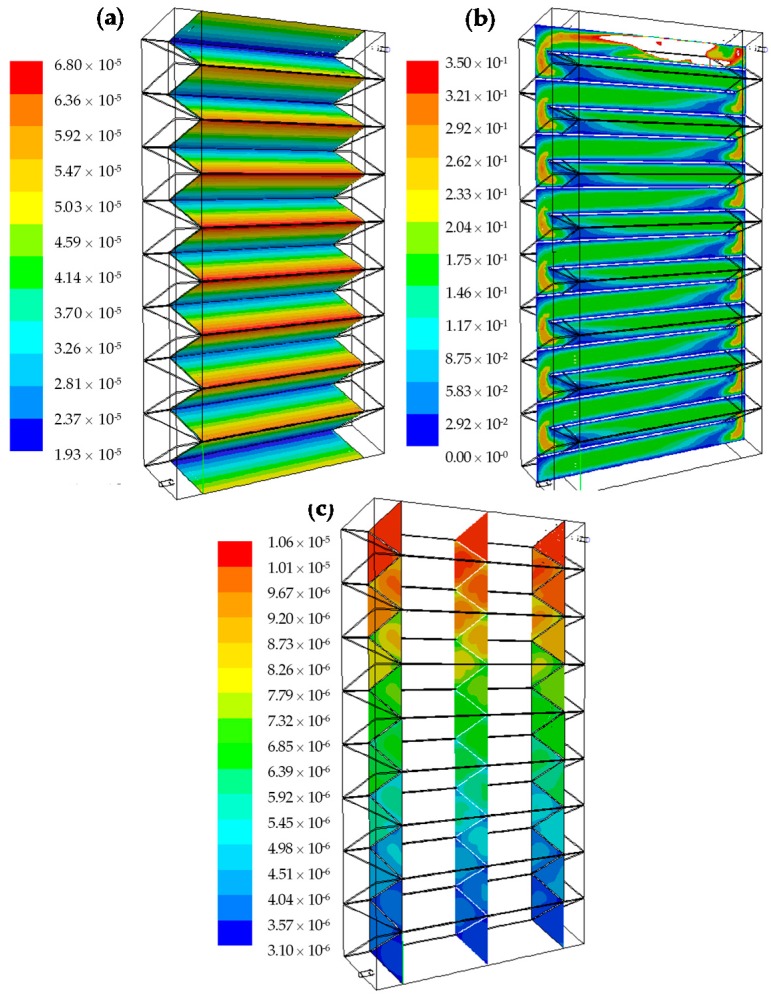
(**a**) LSRPA on the corrugated plate (einstein m^−2^ s^−1^); (**b**) contours of velocity (m/s) in the central plane of the reactor; and (**c**) contours of molar HCHO fractions.

*Radiation Field:* The absorbed radiation on one side of the corrugated plate is presented in Figure 5a. It can be seen that the absorbed radiation is higher at the top than at the bottom of the channels, despite the reflection of the coated surface. In addition, the central channel looks more “illuminated” while the channels towards the extremes receive less radiation, because of the edge effect of the lamps.

*Velocity field:* Figure 5b shows how the velocity profiles develop to reach a symmetrical distribution, with maximum velocities at the centroid of the triangular section and minimum in the corners and walls, where the flow is subject to a no-slip shear condition. The change in direction of 180 degrees and a slight narrowing of the passage section between channels causes the acceleration and the asymmetry in the contours of velocity at the beginning of each channel.

*Concentration field:* Figure 5c depicts a typical output of the pollutant concentration inside the reactor. HCHO concentration field shows a decreasing concentration along the reactor pathway. The effect of the catalytic walls along each channel is noticeable: the concentration of pollutant is maximum at the triangle’s center, in the bulk, and decreases toward the walls where the reaction takes place. It is also clear that the change in direction from one channel to the other acts as a static mixer, renewing the concentration profile of HCHO. This mixing between each channel renews the concentration profile, smoothing the gradients and allowing the arrival of the pollutant to the reactive walls.

Five operating condition sets were simulated, using different inlet HCHO concentrations, water vapor concentrations and radiation levels. The five sets of conditions were experimentally tested in the bench scale photocatalytic reactor. Pollutant conversions from the model simulations were contrasted to the experimental results; good agreement was found between simulation results and experimental data. The root mean square error, based on the experimental and predicted HCHO conversions, was lower than 4%.

### 3.3. Bench Scale Reactor Optimization

The ultimate output of the proposed methodology (Figure 1) is an optimized reactor. The optimization step aims at finding the best configuration given certain restrictions. In order to evaluate different reactor configurations regarding overall conversion of a certain pollutant, some of the parameters that have been employed are: the quantum yields [25,37], the photonic efficiencies [23,38] and the electrical energy per order or per unit mass [39]. A different parameter was also proposed: the photochemical thermodynamic efficiency factor [40].

Here, we use the concept of multiple independent efficiencies in series contributing to the overall reactor performance [14]. This approach allows the identification of reactor design advantages and drawbacks, offering useful tools for subsequent optimization.

The global efficiency of a photocatalytic process can be defined as the product of individual efficiencies, according to:

(12) $$\eta_{T}=\prod_{i}\eta_{i}=\;\eta_{ele}\cdot \eta_{inc}^{out}\cdot \eta_{inc}^{in}\cdot \eta_{abs}\cdot \eta_{rxn}$$

These efficiencies are linked by the output spectral power of the lamps, the transmittance of the windows material and the spectral absorption of the immobilized catalyst. In fact, the range of wavelengths in which the integration of properties is performed determines the coupling among the different efficiencies.

The electrical efficiency: $\eta_{ele}=P_{L}/P$ represents the capacity of the radiation source of converting electrical energy (actually consumed power, P) into emitted radiant power ($P_{L}$) in the desired wavelength range [41]. This parameter has a fixed value for a given brand and model of lamp.

The outer geometrical incidence efficiency represents the ratio of emitted photons that effectively reach the reactor windows. The possibility of occurrence of such event may be called $\eta_{inc}^{out}$ and defined by:

(13) $$\eta_{inc}^{out}\;=\;\frac{\int_{A_{W}}\int_{\lambda }q_{\lambda ,W}^{out}d\lambda dA}{\int_{\lambda }P_{\lambda ,L}d\lambda }\;=\;\frac{\langle q_{w}^{out}\rangle A_{w}}{P_{L}}$$

It is a relative optical parameter between the radiation source and the reactor exterior. The reactor geometry and the lamp arrangement to irradiate the reactor are the only parameters determining this efficiency. The definition of the denominator in Equation (13) must take into account the number of lamps and the presence of reflectors.

Next, we have the inner incidence efficiency. A fraction of the photons that get to the reactor window is transmitted; once inside, they can either reach a catalytic surface or not. The probability of a radiation ray leaving the inner side of the reactor window to get to a catalytic area may be defined as:

(14) $$\eta_{inc}^{in}\;=\;\frac{\langle q_{inc}\rangle A_{cat}}{\langle q_{w}\rangle A_{w}}$$

The internal configuration of the reactor may allow the enhancement of this efficiency by surface to surface radiative interaction through reflection. Thus, the optical properties of the support material are essential because transmittance or reflection may have a positive or negative impact depending on the shape and configuration of the reactor.

Ultimately, the radiation that reaches the catalyst surface may be absorbed or reflected, giving place to another parameter, $\eta_{abs}$, the absorption efficiency.

(15) $$\eta_{abs}\;=\;\frac{\langle e^{a,s}\rangle }{\langle q_{inc}\rangle }$$

which represents the ratio between absorbed and incident energy on the catalytic surface. It is independent of the external reactor geometry, but an intrinsic property of the photocatalyst nature.

Once the catalyst had absorbed radiation, the reaction efficiency is defined as:

(16) $$\eta_{rxn}\;=\;\frac{\langle r_{\sup}\rangle }{\langle e^{a,s}\rangle }$$

The reaction efficiency can be regarded as the moles of pollutant units eliminated per absorbed energy (einsteins) over the catalyst surface.

#### Optimal Folding Angle of the Corrugated Reactor

For the sake of optimization, some design and operative variables were kept constant: the total gas flow rate $Q_{g}$ and the pollutant concentration $C_{i,0}$ to be reduced; the radiation source; and the reactor volume. The design variable to be optimized in the corrugated wall reactor was then the folding angle.

**Figure 6:**
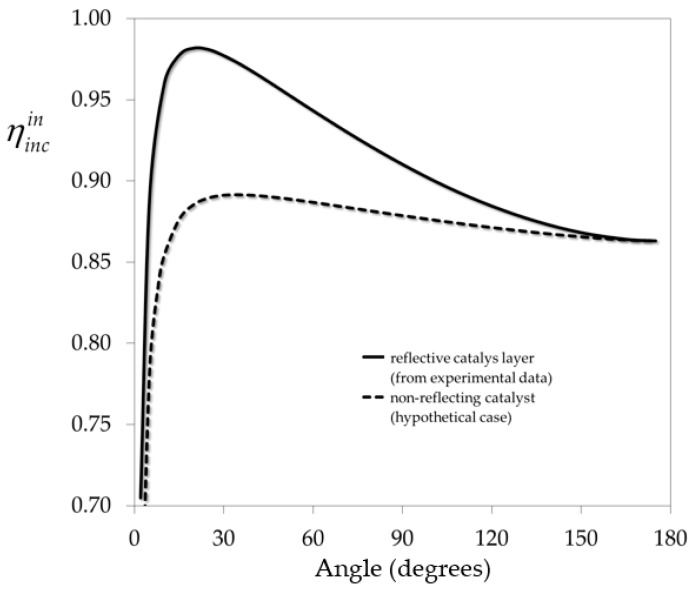
Inner incidence efficiency as a function of folding angle. Reprinted with permission from Passalía, C.; Alfano, O.M.; Brandi, R.J. Optimal design of a corrugated-wall photocatalytic reactor using efficiencies in series and computational fluid dynamics (CFD) modeling. *Ind. Eng. Chem. Res.* **2013**, *52*, 6916–6922. Copyright 2013 American Chemical Society [14].

The influence of the folding angle on the inner incidence efficiency is depicted in Figure 6. It can be seen that this parameter shows an optimum value at angles below 30 degrees. For the case with no reflection (hypothetical), the optimum is lower than that of the real case (with reflection). The fraction of reflected radiation clearly enhances the capabilities of this corrugated wall configuration.

A study was performed by simulation of four folding angles (φ): 97.2, 59.1, 31.6 and 15.2 degrees. Table 3 summarizes the results for the four folding angles. The simulations were performed in the academic version of a CFD software (Fluent 12.1) and the main output of them was the overall pollutant conversion.

The second column in Table 3 contains the absorbed energy relative to the entering radiation through the reactor window; as can be seen, it gets higher when the catalyst surface is almost normal to the radiation source. The third column shows that the averaged superficial reaction rate increases almost five-fold when changing the angle from 15.2 to 97.2 degrees; this is due to the linear dependence of the reaction rate with the LSRPA. The fourth column in Table 3 presents the reaction efficiency, derived indirectly from the previous columns; as can be seen, $\eta_{rxn}$ has a smooth trend to decrease when the folding angle is increased. This behavior is based on the simultaneous increase in the relative absorbed energy and the averaged reaction rate.

The results presented in Table 3 indicate the presence of an optimum between the closest angles simulated, i.e., 15 and 30 degrees. This is consistent with the maximum found for the inner incident efficiency (Figure 6).

## 4. Conclusions

An integral design methodology has been proposed and applied to scaling-up and optimization of photocatalytic wall reactors employed in air pollution control. Starting from a kinetic study in a laboratory scale reactor and the evaluation of the absorbed radiation, a bench scale reactor could be successfully modeled and optimized. The proposed methodology shows a systematic approach based on engineering concepts and can be used to design and optimize any photocatalytic wall reactor provided that the balances involved, including radiation, can be solved by a mathematical model.

## Figures and Tables

**Table 1:** Main characteristics of reactors and operating conditions.

Description	Laboratory Scale Reactor	Bench Scale Reactor
Configuration	Flat plate	Corrugated plate
Main dimensions	8 (W) × 8 (L) × 0.4 (T) cm	20 (W) × 30 (L) × 3 (T) cm
Reactor volume	25.6 cm^3^	1800 cm^3^
Catalytic surface area	64 cm^2^	1843.5 cm^2^
Number of lamps	5	10
Flow Rate	1–3.5 L/min	
Inlet HCHO concentration	5–35 ppmv	
Relative Humidity	10–75%	
Maximum radiation	8.94 × 10^−5^ einstein m^−2^ s^−1^	
Radiation levels	16%, 26%, 60%, 100%	

**Table 2:** Kinetic pathway for formaldehyde (HCHO) photo-oxidation.

Step	Reaction	Reaction
Initiation	1	$TiO_{2}\;+\;h\nu \;\to \;TiO_{2}\;+\;e^{-}\;+\;h^{+}$
Recombination	2	$e^{-}\;+\;h^{+}\;\to \;\mathrm{Heat}$
O•H generation	3	h+ + OH− → O•H
Electron trapping	4	e− + O2 → O2−•
HCHO Oxidation	5	HCHOads + O•H → C•HO + H2Oads
	6	C•HO + O•H → HCOOHads
Ending reactions	7	HCOOHads + O•H → Products
	8	M + O•H → Products
Adsorption equilibria	9	$HCHO\;+\;\mathrm{Site}\;⇄HCHO_{\mathrm{ads}}$
	10	$H_{2}O\;+\;\mathrm{Site}\;⇄H_{2}O_{\mathrm{ads}}$
Site balance	11	${\mathrm{Site}|}_{\mathrm{Total}}\;=\;{\mathrm{Site}|}_{HCHO}\;+\;{\mathrm{Site}|}_{H_{2}O}\;+\;\mathrm{Site}$

**Table 3:** Results of efficiencies for different folding angles. Adapted with permission from Passalía, C.; Alfano, O.M.; Brandi, R.J. Optimal design of a corrugated-wall photocatalytic reactor using efficiencies in series and computational fluid dynamics (CFD) modeling. *Ind. Eng. Chem. Res.* **2013**, *52*, 6916–6922. Copyright 2013 American Chemical Society [14].

Angle	$\langle e^{a,s}\rangle /\langle q_{w}\rangle$	$\langle r_{sup}\rangle \times 10^{11}$	$\eta_{rxn}\times 10^{3}$	$\eta_{inc}$
(degrees)	-	(mol cm^−2^ s^−1^)	(mol Einstein^−1^)	-
15.2	0.078	1.04	9.24	0.978
31.6	0.155	2.09	9.29	0.976
59.1	0.271	3.50	8.93	0.944
97.2	0.395	4.74	8.28	0.903

## Nomenclature

$a_{v}$	external catalytic surface area per unit volume (cm^−1^)
$A_{c}$	cross sectional area (cm^2^)
$A_{cat}$	photocatalytic area (cm^2^)
C	molar concentration (mol cm^−3^)
$e^{a,s}$	local superficial rate of photon absorption, LSRPA (einstein cm^−2^ s^−1^)
*H*	height (cm)
*I*	specific radiation intensity (einstein cm^−2^ s^−1^ sr^−1^)
$I^{0}$	specific radiation intensity on the lamp surface (einstein cm^−2^ s^−1^ sr^−1^)
$k_{m}$	gas phase mass transfer coefficient (cm s^−1^)
*L*	length (cm)
*P*	emission power (einstein s^−1^)
*q*	radiation flux (einstein cm^−2^ s^−1^)
*Q*	volumetric gas flow rate (cm^3^ s^−1^)
*R*	homogeneous reaction rate (mol cm^−3^ s^−1^)
*r*	heterogeneous reaction rate (mol cm^−2^ s^−1^)
$r_{g}$	local superficial rate of electron-hole pair generation (mol cm^−2^ s^−1^)
*R_Li_*	lamp radius (cm)
*T*	reactor thickness (cm)
$T_{\lambda }$	spectral transmittance (dimensionless)
$v_{z}$	axial velocity (cm s^−1^)
*W*	reactor width (cm)
x,y,z	rectangular coordinate system (cm)
$\underline{x}$	position vector (cm)
*X*	conversion (dimensionless)
*Greek letters*	
α	kinetic parameter (cm^3^ einstein^−1^)
η	efficiency (dimensionless)
κ	adsorption constant (cm^3^ mol^−1^)
λ	wavelength (nm)
ρ	gas density (g cm^−3^)
$\underline{\underline{\tau }}$	viscous stress tensor
ϕ,θ	spherical coordinates (rad)
Φ	primary quantum yield (mol einstein^−1^)
$\bar{\Phi }$	wavelength averaged primary quantum yield (mol einstein^−1^)
*Subscripts*	
ads	indicates adsorbed species
abs	relative to radiation absorption
*F*	relative to formaldehyde
*F_i_*	relative to radiation neutral filter
*Li*	relative to lamp i
*W*	relative to water
*W_i_*	relative to reactor window
λ	indicates wavelength dependence
*exp*	relative to experimental data
*mod*	relative to model
*Superscripts*	
*in*	relative to reactor inlet
*out*	relative to reactor outlet
*Special symbols*	
〈〉	denotes an averaged property
$\underline{}$	denotes a vector
